# Supplementary material for: Intravital imaging strategy FlyVAB reveals the dependence of Drosophila enteroblast differentiation on the local physiology
Source: Commun Biol. 2021 Oct 25;4:1223. doi: 10.1038/s42003-021-02757-z (PMC8546075; doi:10.1038/s42003-021-02757-z)
Supplement: Supplementary file 2 — Supplementary Information [file 42003_2021_2757_MOESM2_ESM.pdf]

Supplementary materials:

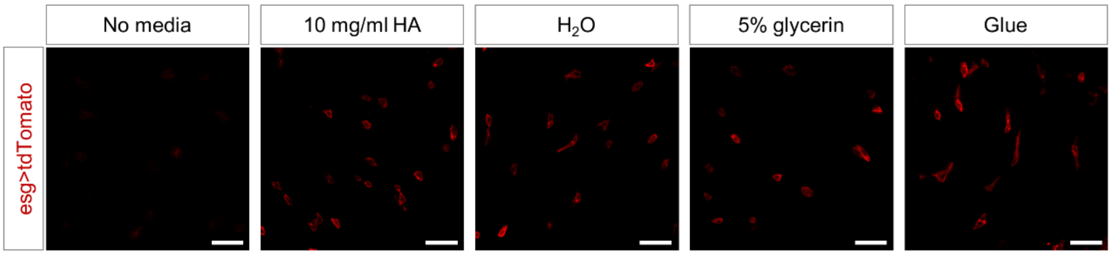

Supplementary Figure 1. The test of mounting media. The adult *Drosophila* midgut was visible using 10 mg/ml HA, H_2O, 5% glycerin or glue. Genotype: *esg-Gal4 10×UAS-myr:tdTomato*. All scale bars are 20 µm.

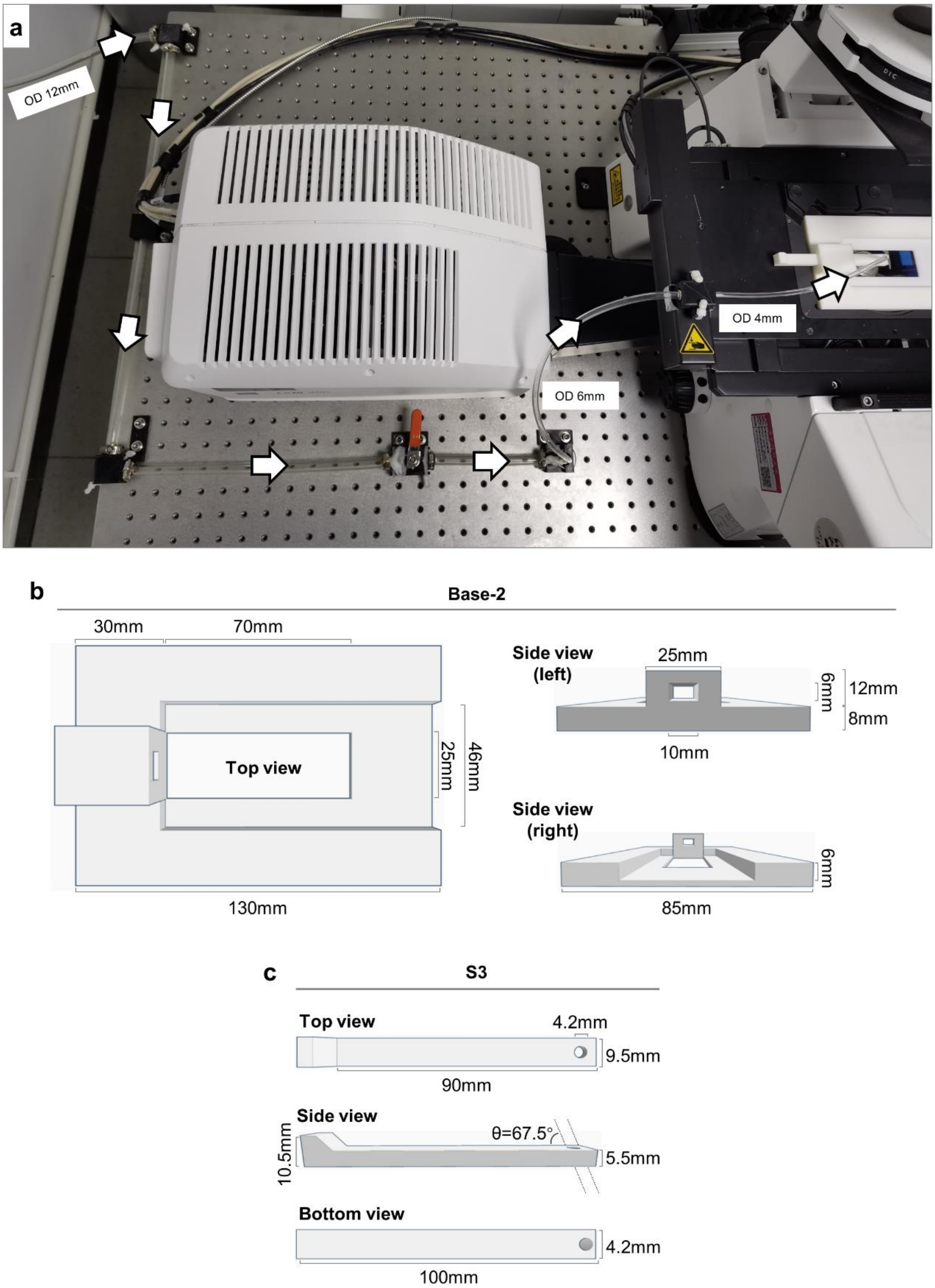

Supplementary Figure 2. Design of the FlyVAB (P2) and (P3). a Representative image of the FlyVAB (P2) from the top. FlyVAB (P2) was designed as a CO_2 delivery system to perform *Drosophila* anesthesia. The white arrows show the direction of CO_2 flow. The size of three types of PU tubing is indicated. **b, c** Exploded view drawing of the FlyVAB (P3). FlyVAB (P3) consists of the Base-2 (**b**) and S-3 (**c**). At the bottom of the Base-2, a window was present to place the 20× objective of the microscope, and a groove to hold FlyVAB (P1). The removable S3 was placed through a small window on the left side of Base-2. The punching was designed in S3 to hold the PU

tubing. An angle of 67.5 degree was present between the punching and the horizontal line.

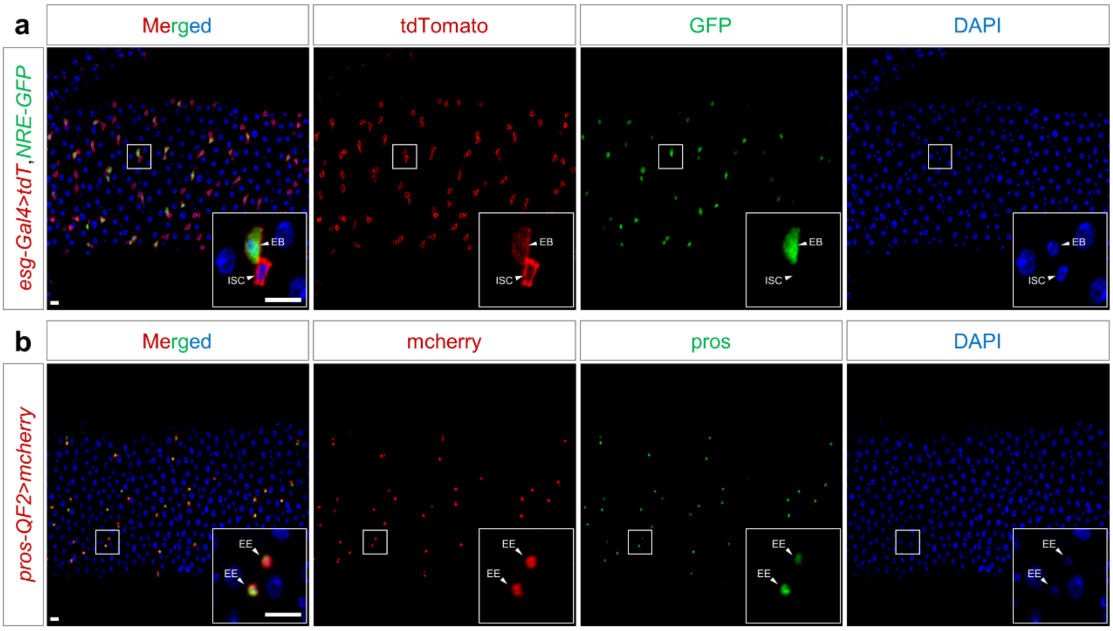

Supplementary Figure 3. **Identification of ISCs, EBs, and EEs in fixed tissues. a** Immunostaining of GFP after dissection at 5 days after eclosion. An ISC and an EB are indicated by white arrowheads (the square is enlarged in the lower right corner). **b** Immunostaining of Pros after dissection at 5 days after eclosion. An EE is indicated by a white arrowhead (the square is enlarged in the lower right corner). Genotype in **a**: *esg-Gal4 10×UAS-myr:tdTomato, NRE-GFP*. Genotype in **b**: *pros-QF2 20×QUAS-6xmcherry*. All scale bars are 10 μm in **a-b**.

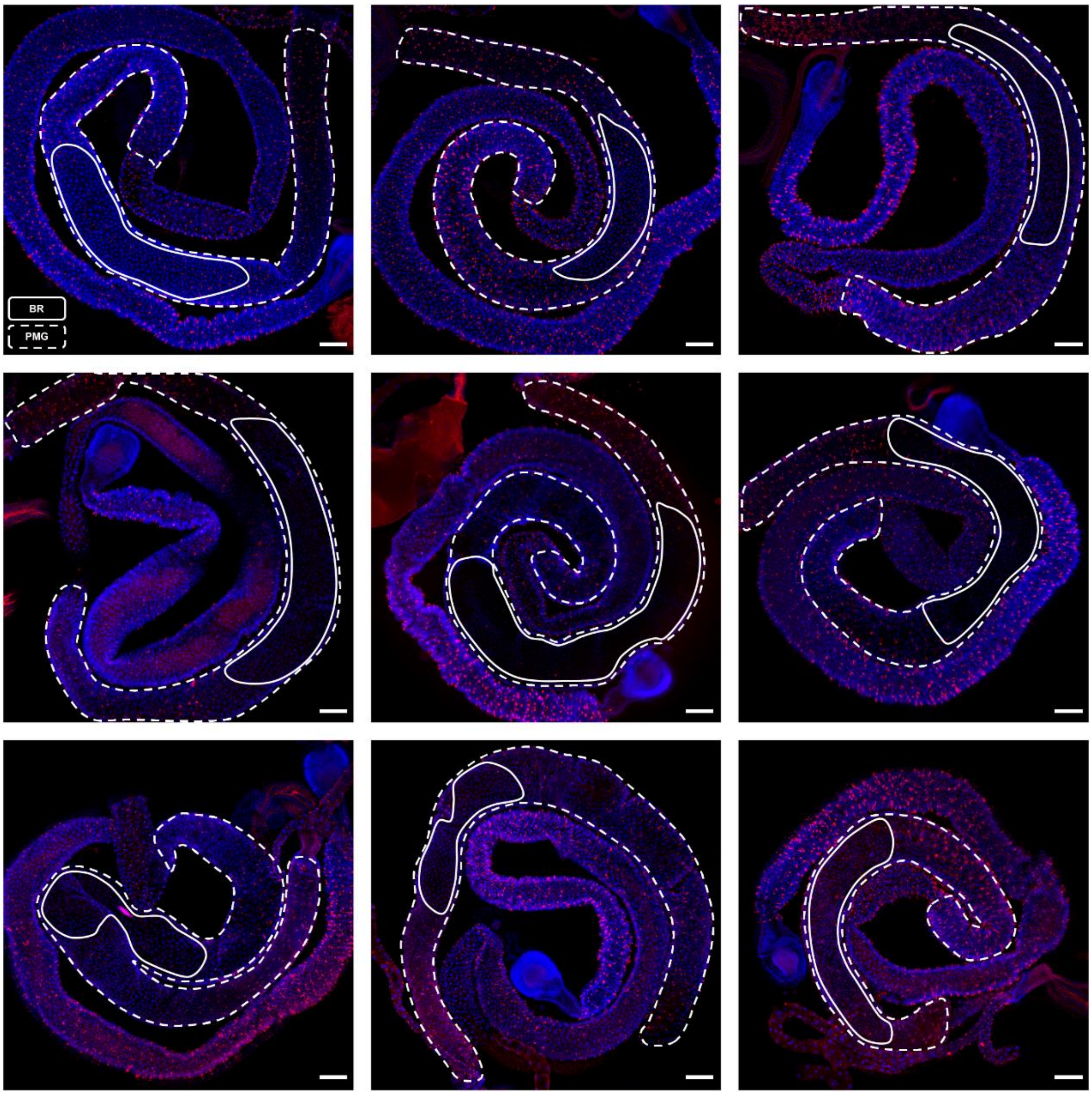

Supplementary Figure 4. Determination of the visualized region in the midgut by photobleaching in nine additional flies. Gut samples were subjected to photobleaching, then dissected, fixed, and stained with DAPI (blue). BR: bleached region (solid line). PMG: posterior midgut (dashed line). Genotype: *esg-Gal4 10×UAS-myr:tdTomato*.

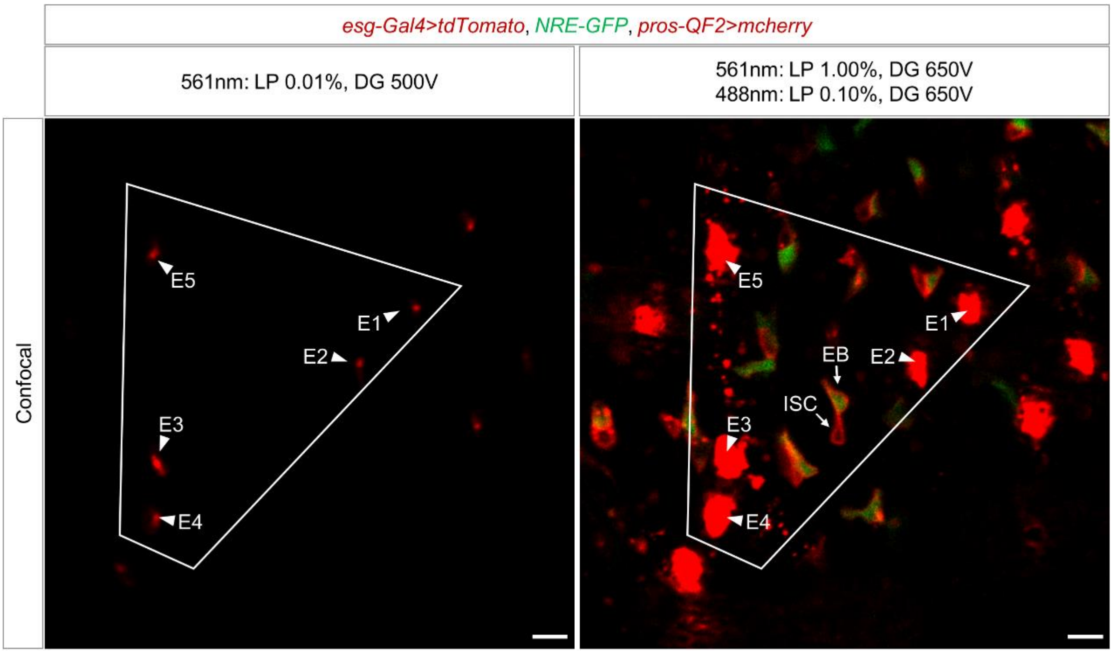

Supplementary Figure 5. Visualization of ISCs, EBs and EEs in the same fly. Low laser power (LP) and low digital gain (DG) under the 561 nm laser line identified only EEs (*pros-QF2>mcherry*, E1-E5, solid line). Normal LP and DG under 488 nm and 561 nm laser line identified ISCs (*esg-Gal4>tdTomato*), EBs (*esg-Gal4>tdTomato, NRE-GFP*), and EEs, but EEs were overexposed. Genotype: *esg-Gal4 10×UAS-myr:tdTomato, NRE-GFP; pros-QF2 20×QUAS-6×mcherry*. All scale bars in a and b are 10 μm.

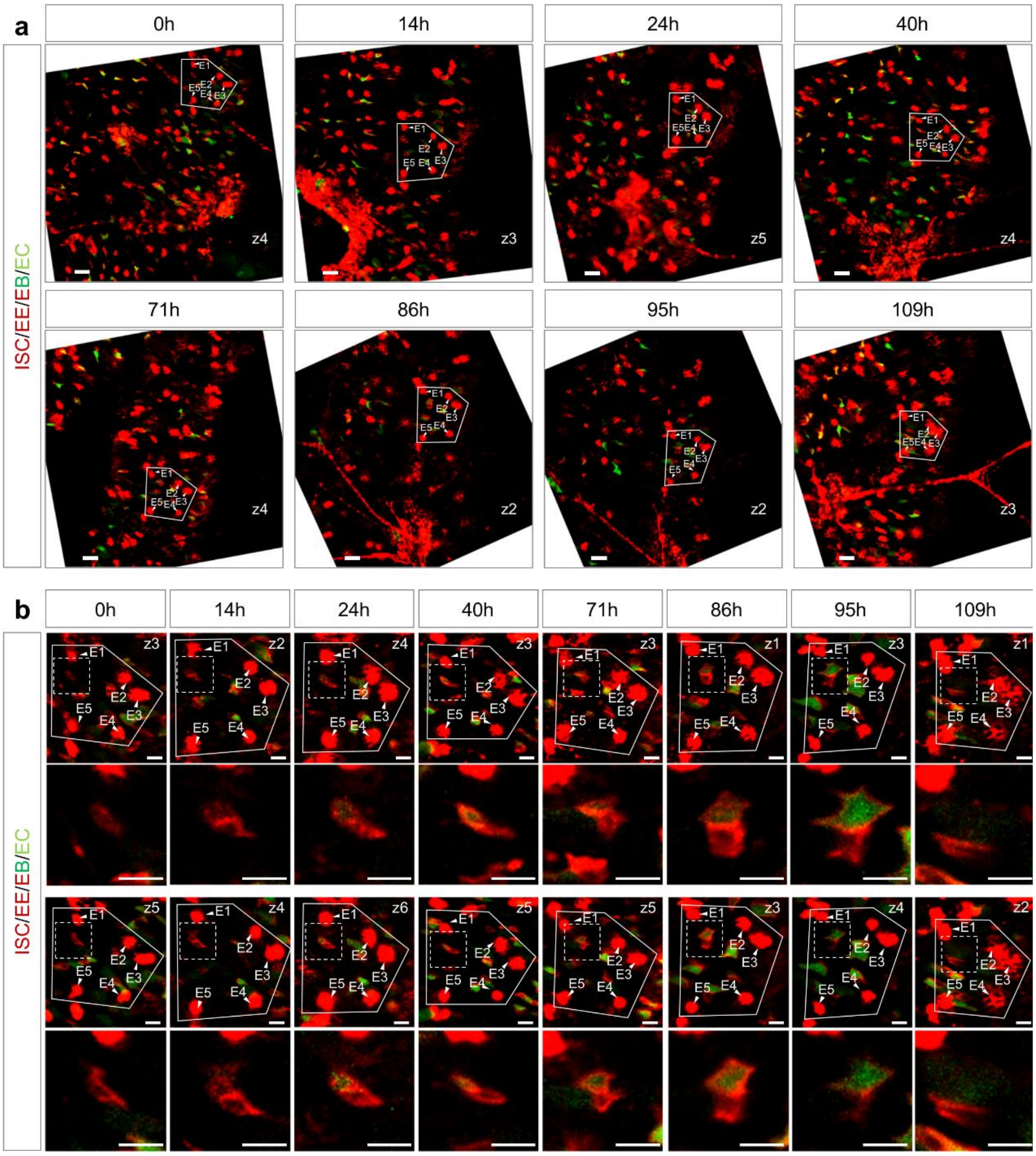

Supplementary Figure 6. Additional images of Figure 5a. a Full view of the images. Individual images were rotated to compare the EE cell pattern (E1-E5, solid line) over time. The z-section of each image is indicated in the lower right corner. Scale bars are 20 µm. **b** Images of two additional z-sections. Z-sections are indicated in the upper right corner. The region of interest (dashed square) at each individual z-section is enlarged in the second and fourth row. Scale bars are 10 µm. Genotype: *esg-Gal4 10×UAS-myr:tdTomato, NRE-GFP; pros-QF2 20×QUAS-6×mcherry*.

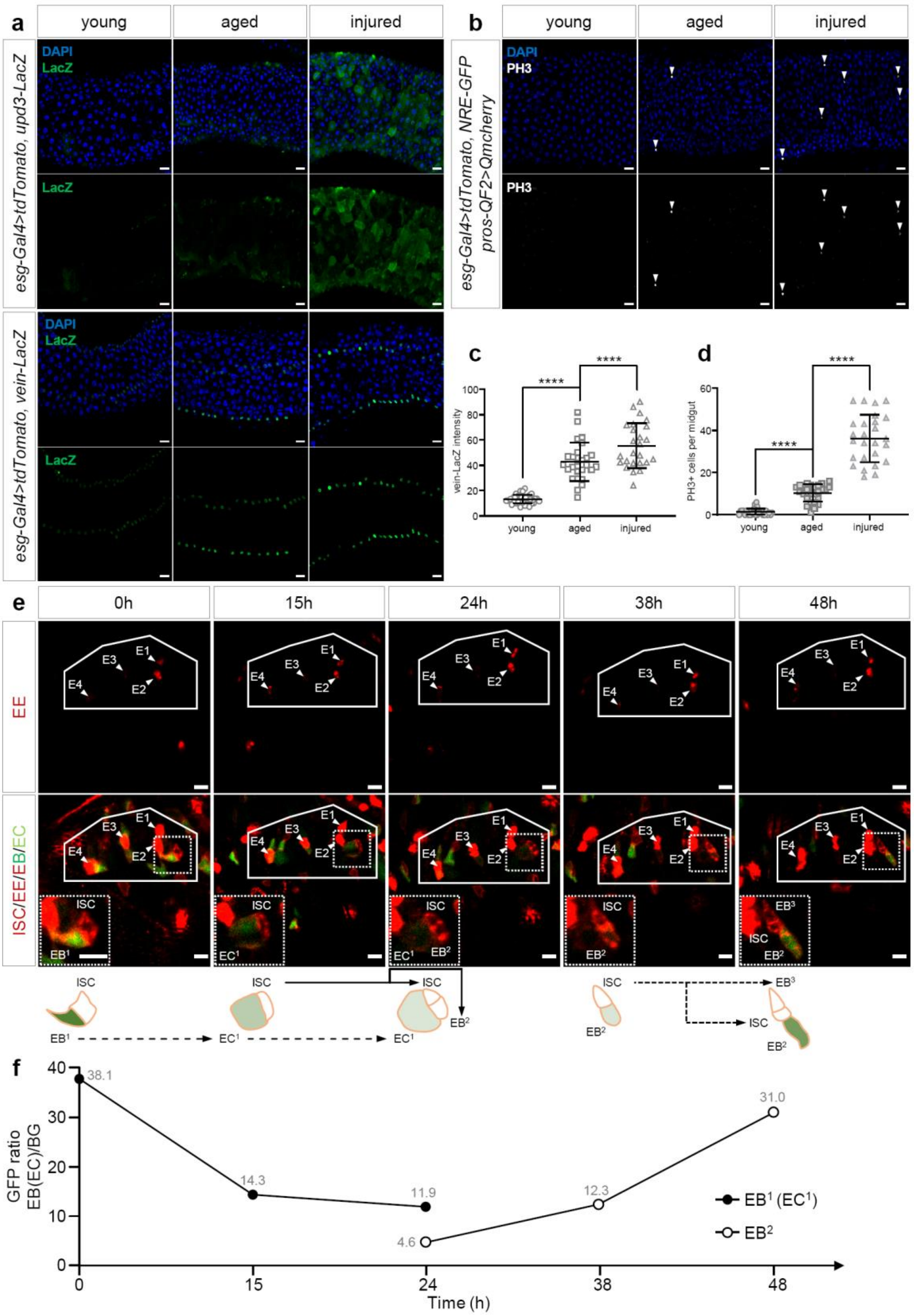

Supplementary Figure 7. 14 days old intestines were in a mild hyperplastic status and *in vivo* tracking of ISCs and EBs upon local EC renewal. **a** Representative images of *upd3-lacZ*, *vein-lacZ* staining in young (7 days), aged (14 days) and injured (1 day after bleomycin treatment) flies. Genotype: *esg-Gal4 10×UAS-myr:tdTomato/upd3-lacZ or vn-lacZ*. **b** Representative images of PH3 staining in young, aged and injured flies. White arrowhead: PH3+ cell. Genotype: *esg-Gal4 10×*

UAS-myr:tdTomato, NRE-GFP; pros-QF2 20×QUAS-6×mcherry. **c** Quantification of the *vein-lacZ* intensity in **a**. Results are shown as mean ± SD. **d** Quantification of PH3+ cells per midgut in young, aged and injured flies. Results are shown as mean ± SD. **e** Representative images of ISC division and differentiation with local EC renewal. Using the EE pattern (E1-E4, solid line, first row), an ISC-EB pair (ISC and EB^2, the dashed square is enlarged in the lower left corner) was tracked at 5 time points over 48 h. An EB-EC transition (EB^1 to EC^1) was identified over 24 h. The ISC divided once to generate an ISC and an EB^2 along with the EC^1 generation. Shortly afterwards, ISC divided again to generate an ISC and an EB^3. Genotype: *esg-Gal4 10×UAS-myr:tdTomato, NRE-GFP; pros-QF2 20×QUAS-6×mcherry*. **f** Quantification of GFP ratio in EB^1 (EC^1) and EB^2. The value of GFP ratio is indicated at each time point. Scale bars are 20 μm in **a** and **b**, 10 μm in **e**.
