# Supplementary material for: Intravital imaging strategy FlyVAB reveals the dependence of Drosophila enteroblast differentiation on the local physiology
Source: Commun Biol. 2021 Oct 25;4:1223. doi: 10.1038/s42003-021-02757-z (PMC8546075; doi:10.1038/s42003-021-02757-z)
Supplement: Supplementary file 3 — Description of Additional Supplementary Files [file 42003_2021_2757_MOESM3_ESM.pdf]

Description of Additional Supplementary Files

File name: Supplementary Movie 1.
Description: Video protocol of the FlyVAB method.

File name: Supplementary Movie 2.
Description: Flies that were subjected to one-time squeezing had a similar amount of deposits.

File name: Supplementary Movie 3.
Description: Timelapse movie of calcium signal in 10 min. Red, esg-Gal4 drived tdTomato. Green, esg-Gal4 drived GCaMP.
